# Platelets transport β-amyloid from the peripheral blood into the brain by destroying the blood-brain barrier to accelerate the process of Alzheimer's disease in mouse models

**DOI:** 10.18632/aging.202662

**Published:** 2021-03-05

**Authors:** Tong Wu, Lizhi Chen, Lingqi Zhou, Jie Xu, Kaihua Guo

**Affiliations:** 1 Department of Anatomy and Neurobiology, Zhongshan School of Medicine, Sun Yat-Sen University, Guangzhou, P.R. China; 2 Department of Science and Education, Guangdong Second Provincial General Hospital, Guangzhou, P.R. China

**Keywords:** Alzheimer’s disease, platelets, blood-brain barrier, aspirin, β-amyloid

## Abstract

Extracellular aggregation of the β-amyloid (Aβ) peptide into toxic multimers in the brain is a prominent event occurring in the pathogenesis of Alzheimer’s disease (AD), and a large amount of Aβ in the blood is derived from platelets. Thus, we speculated that platelets may play an important role in the process of AD. We first investigated the changes in platelet Aβ secretion with age. Then, we injected platelets from aged amyloid precursor protein APP/PS1 mice into young C57 mice and assessed their memory capacity along with their brain and peripheral blood Aβ expression levels. The Aβ content in mouse platelets increased with age. Exogenously aged APP/PS1 platelets changed the permeability of the blood-brain barrier *in vitro*, accelerating Aβ deposition in the brain and increasing the Aβ content in peripheral blood, leading to learning and memory deficits in the recipient mice. Subsequently, aspirin was administered to mice as an inhibitor of platelet activation, which effectively alleviated these toxic processes. Finally, we chose an *in vitro* blood-brain barrier model to explore the possible cytotoxicity of these platelets.

## INTRODUCTION

Alzheimer’s disease (AD) is a neurodegenerative disease clinically characterized by memory deterioration and functional impairment that has grave consequences for elderly individuals [1, 2]. The typical pathological features of AD are senile plaques formed by the precipitation of extracellular β-amyloid (Aβ), neurofibrillary tangles formed by intracellular tau protein aggregates, and the loss of many neurons [3]. Amyloid precursor protein (APP) is cleaved into different Aβ peptides, among which the 40 amino acid peptide Aβ1-40 and the less soluble 42 amino acid peptide Aβ1-42 are the most abundant forms and considered two of the most important peptides in the development of AD [4–6]. Many studies have explored Aβ deposition in the brain, but few have reported the role of Aβ derived from the circulatory system that enters into the nervous system [7, 8]. Thus, the pathogenesis of AD remains to be further elucidated.

Circulating platelets secrete more than 90% of Aβ in peripheral blood, where Aβ1-40 is the predominant form [9–11]. Platelets are rich in APP and the proteases required for Aβ metabolism. Moreover, the concentration of platelet APP isoforms is comparable to the concentration of APP isoforms in the brain, providing valuable insights for studies of peripheral treatments for AD [12, 13]. Our previous studies revealed an association between platelets and Aβ metabolism [14, 15]. Activated platelets secrete proinflammatory mediators, which may lead to amplified peripheral inflammation and endothelial senescence [16, 17]. All human platelets produce Aβ, but not everyone will suffer from AD as they age. The causes of this difference are worth exploring.

Recently, circulating Aβ has been shown to cross the blood-brain barrier (BBB) [18]. The BBB is a highly selective biological barrier, mainly composed of microvascular endothelial cells, astrocytes and pericytes that prevents the entry of various harmful substances into the brain from the blood [19]. Most macromolecular substances do not pass through the BBB, and only fat-soluble substances and substances with a relatively small molecular mass or diameter cross through it [20, 21]. Combined with the biochemical properties of platelets, we hypothesized that platelets play an important role in transporting peripheral Aβ across the BBB, and Aβ deposits in the brain might become a crucial factor in the pathogenesis of AD.

A population-based retrospective cohort study indicated that daily use of aspirin (acetylsalicylic acid, ASA) reduced the risk of developing AD in patients with type 2 diabetes, and another study found that it reduced the secretion of Aβ from platelets [22, 23]. ASA, a traditional antipyretic analgesic, was found to inhibit platelet aggregation, and has been applied to prevent and treat ischemic heart disease and cerebral thrombosis [24]. Thomas and Harris et al. reported that ASA inhibits Aβ aggregation *in vitro*, and long-term use of non-steroidal anti-inflammatory drugs such as ASA may reduce the risk of AD [25, 26]. ASA inhibits platelet aggregation by inhibiting cyclooxygenase (COX) activity, and may also improve synaptic dysfunction by affecting COX-dependent mechanisms to improve the symptoms of AD [27, 28]. Overall, these studies peaked our interest as to whether ASA would reduce the Aβ deposition in the brain and improve cognitive and learning abilities.

In this study, AD model mice, namely, SAM mice and APP/PS1 mice, were used to investigate the relationship between platelets and the AD process in aged mice. We designed experiments by injecting platelets to explore their possible effects on the AD process, while an *in vitro* BBB model was chosen to explore the underlying mechanisms.

## MATERIALS AND METHODS

### Drugs and reagents

The aspirin (99% purity) used for the experiment was purchased from Sigma-Aldrich (St. Louis, MO, USA). All cell culture reagents were purchased from Gibco (Grand Island, NY, USA). The Aβ1-40, Aβ1-42 and tau ELISA kits were purchased from Wuhan Elabscience Biotechnology Co. Ltd. (Wuhan, China). The Cell Counting Kit-8 and Hoechst 33342 staining solution were purchased from Dojindo Laboratory (Japan). Antibodies specific to 6E10 and Iba1were purchased from CST (Danvers, MA, USA).

### Animals and cell culture

APP/PS1 mice and C57BL/6 mice were purchased from the Model Animal Research Center of Nanjing University (Nanjing, CHN). SAMP8 (senescence-accelerated prone 8) mice and SAMR1 (senescence-accelerated resistant 1) mice were purchased from Charles River (Beijing, China). Mice were housed in an SPF environment with light and dark available for 12 hours each day, a temperature of 22±2° C, and humidity of 55±5%, and were fed a standard rodent provender. Mice had free access to water and food. All experiments were conducted according to the Guidance for Animal Care and Use in China. All protocols were approved by the Animal Ethics Committee of Sun Yat-sen University (SYSU-IACUC-2019-B990).

B End.3 and HT22 cell were purchased from the Cell Bank of the Chinese Academy of Sciences (Shanghai, CHN). All cells were cultured in DMEM supplemented with 10% fetal bovine serum, and incubated in incubator with 5% CO_2_ at 37° C.

### Preparation of platelets and plasma

All the mice were anesthetized by injecting sodium pentobarbital (1 mg/10 g body weight), and platelets were obtained as previously reported [14]. Blood samples (approximately 1 mL) were acquired from the suborbital vein and placed in centrifuge tubes containing 40 μL of EDTA (15 g/L). Then, platelet-rich plasma (PRP) was attained by centrifugation (200 g, 10 min). Thereafter, the platelet pellet was obtained by another centrifugation (1000 g, 10 min) of the PRP. The plasma without platelets was placed into another tube. The samples described above were stored in a liquid nitrogen tanker or -80° C freezer until use in subsequent experiments.

### Groups and administration

Three-, six-, ten-, fifteen-month-old APP+/PS1+ mice and APP-/PS1- mice were used to provide platelets and plasma for the following experiments. Ten-week-old C57BL/6 mice were randomly divided into 12 groups, with 4 mice in each group (2 of each sex) separated into 2 cages by sex. The animals were injected with platelets or plasma virtually free of platelets from the mice described above through tail vein once a week, four times in total. The platelet and plasma were diluted with physiological saline to 150 μL for injection.

**Figure 1:**
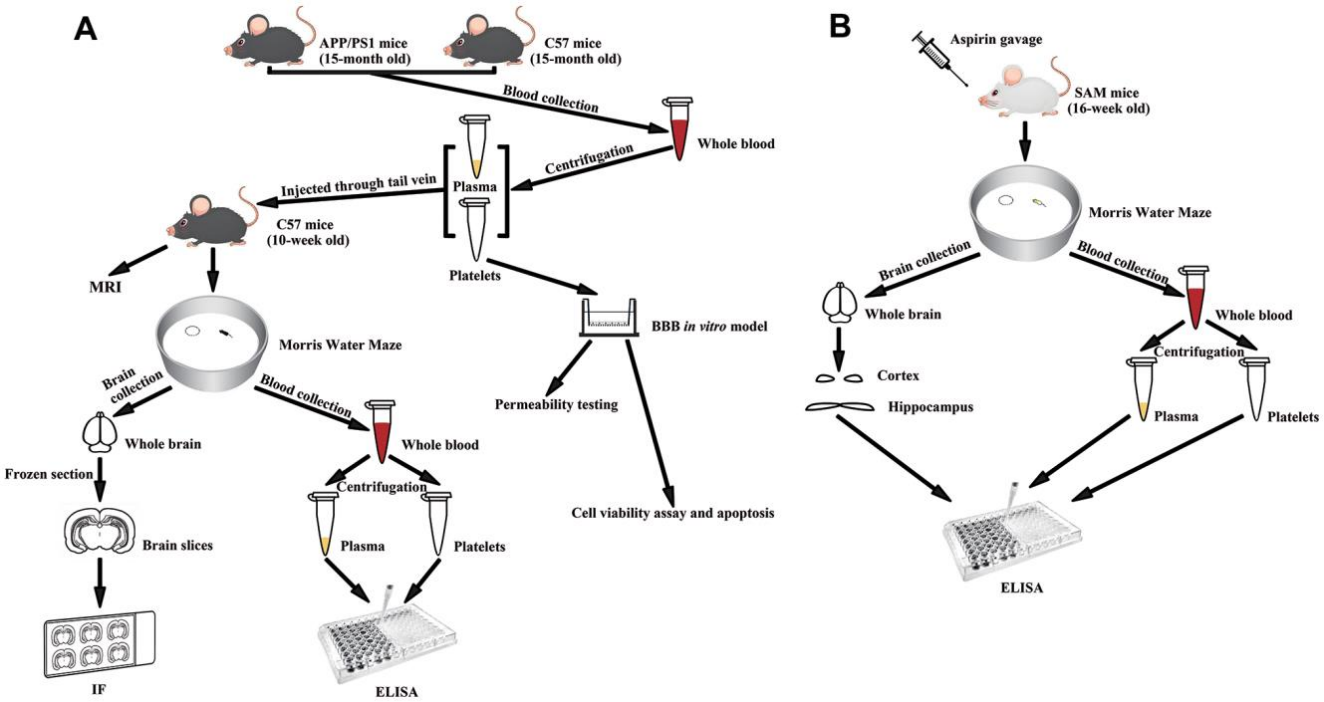
**Experimental diagram.** (**A**) Experimental flowchart of APP/PS1 mice injected with senescent platelets through tail vein. (**B**) Experimental flowchart of SAM mice given aspirin by gavage.

Fifteen-month-old APP+/PS1+ mice and fifteen-month-old C57BL/6 mice were used to provide platelets and plasma for the following experiment. Ten-week-old C57BL/6 mice were randomly divided into 5 groups, with 8 mice in each group (4 of each sex) separated into 2 cages by sex. They were injected with platelets (C57+APP/PS1 platelets) or plasma without platelets (C57+APP/PS1 plasma) from 15-month0old APP+/PS1+ mice; or platelets (C57+C57 platelets) or plasma without platelets (C57+C57 plasma) from 15-month-old C57BL/6 mice; or physiological saline (C57+NS) through tail vein as mentioned above. The experimental design is shown in Figure 1A.

Sixteen-week-old SAMP8 mice were randomly divided into 2 groups, with 16 mice in each group (8 males and 8 females) separated into 4 cages by sex. Sixteen-week-old SAMR1 mice were randomly divided into 2 groups, with 6 mice each group (3 of each sex) separated into 2 cages by sex. ASA (SAMP8/SAMR1 + ASA) or the same volume of water (SAMP8/SAMR1 + ddH_2_O) was administered to the mice by gavage daily for 9 weeks. Ten SAMP8 mice and 6 SAMR1 mice were randomly selected for Morris water maze (MWM) experiments. Blood samples from all SAMP8 mice were used to measure Aβ1-40 and Aβ1-42 levels in platelets and plasma, and 10 blood samples were randomly selected to measure tau levels in platelets and plasma. Ten brain tissue samples were randomly selected to measure Aβ1-40, Aβ1-42 and tau levels in the hippocampus and cortex. The experimental design is shown in Figure 1B.

### Morris water maze test

The MWM has been used to evaluate spatial cognition, learning and memory. The MWM test was conducted using a round white pool with a diameter of 120 cm and a depth of 50 cm. The pool was filled to a depth of 25 cm with water made opaque with titanium dioxide. The temperature of water was maintained at approximately 22° C. The escape platform was a Plexiglas circle with a diameter of 6 cm located in the center of the first quadrant of the pool, 0.5-1 cm underneath the water surface. The MWM consisted of consecutive spatial learning training days and one probe trial day. The spatial learning training days included four successive trials per day in which the platform position remained at the same site, and the experiments were sequentially performed from the middle of the four quadrant edges. Probe trials were performed 24 h after the last trial. The escape times and trajectories of all mice were recorded by a camera mounted on the ceiling above the pool for the memory consolidation assessment [29].

### Collection of brain tissues from mice after behavioral testing

All mice were anesthetized with sodium pentobarbital (1 mg/10 g body weight). Platelets and plasma were obtained as mentioned above, and then animals were perfused through the left ventricle with a saline solution. A portion of the mouse brains was separated immediately to dissect the hippocampus and cortex for protein measurements. The other mice were subsequently perfused with 4% paraformaldehyde. Their brains were removed directly, fixed with 4% paraformaldehyde overnight, and dehydrated with a 30% sucrose solution at 37° C. Then, the brains were embedded in OCT (optimal cutting temperature compound), coronally sectioned into 30 μm slices with a freezing microtome (Thermo NX50), and the frozen sections were prepared for immunofluorescence and immunohistochemical staining.

### Enzyme-linked immunosorbent assay (ELISA)

Aβ1-40, Aβ1-42 and tau levels were determined using ELISAs, and Aβ and Tau levels were normalized to the total protein content in the samples. The protocols of the ELISA kit were followed to complete the remaining steps. The optical densities in each well were measured at 450 nm using a microplate reader.

### *In vitro* transwell BBB model

B End.3 and HT22 cells were purchased from the Cell Bank of Typical Culture Preservation Committee of the Chinese Academy of Sciences. Cells were maintained in a cell incubator (37° C and 5% CO_2_).

The Transwell BBB model was established as previously reported. Briefly, the b End.3 cells (0.2 mL of 5×10^5 cells/mL) were seeded into the upper chamber of an insert (Transwell, polycarbonate membrane, 0.4 μm pore size, 6.5 mm diameter, 24-well plate, Costar, Corning, NY). Next, 0.8 mL of complete media was added to the lower chamber of each well. The system was incubated for 2 days to allow cells to form tight junctions. Subsequently, the HT22 cells was seeded in the lower chamber of each well and cultured for another day [30]. The model was considered mature when trans-endothelial electrical resistance (TEER) values reached 60 Ω*cm^2^, and the permeability coefficient remained below 0.02 [31].

### Assessment of the permeability of the *in vitro* BBB model

After the model was built, the liquid in the upper chamber was first aspirated and rinsed once with phosphate-buffered saline (PBS). Two hundred microliters of a 0.5% Evans blue solution were then added to the upper chamber of each well, and the media in the lower chamber were replaced with 1 mL of PBS per well, followed by an incubation at 37° C. After an incubation for 0, 10, 20, 30, 40, 50 min, the liquid in the lower chamber was transferred to the enzyme-linked plate, and the luminescence value at 620 nm was measured with a chemiluminescence enzyme-linked enzyme analyzer, while the liquid from the Transwell chamber was used as a blank control. The permeability of the BBB model was calculated using the following formula: 1/PS=1/me-1/mf; Pe=PS/s (me: slope of the experimental sample curve; mf: slope of the blank control curve; s: bottom area of the Transwell chamber).

### Cell viability and apoptosis assays

Cell Counting Kit-8 (CCK-8, Dojindo, Japan) were used to evaluate the viability of b End.3 and HT22 cells incubated with or without platelets. Cells were seeded in 96-well plates and after the administration of different interventions to each group, 10 μL of the CCK-8 solution were added to each well. The optical density of each well were measured with a plate reader at 450 nm.

The Hoechst 33342 staining solution were used to visualize apoptotic cells. After the administration of different interventions to each group, the medium was removed and the plates were washed twice with PBS; Hoechst 33342 staining solution was then added to the wells. After an incubation for approximately 15 minutes in the dark, cells were washed with PBS three times, observed and imaged using a fluorescent microscope.

### Immunofluorescence (IF) staining

First, samples were treated with 70% formic acid at room temperature for 10 minutes if the primary antibodies included 6E10. Samples were then treated with a blocking solution consisting of 10% donkey serum and 0.3% Triton in PBS for 60 minutes. Afterwards, the samples were stained with the primary antibody (1:200) for 16 h at 4° C in the dark to label activated microglia and Aβ deposits. After three washes with PBS, samples were subsequently stained with secondary antibody for 1 h at 37° C. Finally, the sections were stained with a 0.2% DAPI solution, mounted on slides with glycerin and a cover glass, and imaged using a fluorescence microscope.

### MRI

Gadodiamide (Gd-DTPA) has been shown to reduce the longitudinal relaxation rate when it diffuses to the extravascular extracellular region, thus enhancing the T1 signal intensity in the region where the BBB opening has occurred. Gd-DTPA was injected after mouse modeling was completed, and the respiration of the mouse was monitored throughout the procedure. Twelve sequential images were acquired over a time window of approximately 30 min after the Gd-DTPA injection, which depicted the diffusion of the contrast agent into the targeted hippocampus and the surrounding area [32].

### Statistical analysis

All data are presented as means±SEM. SPSS 20 and GraphPad Prism 8 software were used to plot and analyze data. Mauchly’s test of sphericity combined with the Greenhouse-Geisse test were used to analyze the MWM data. One-way analysis of variance (ANOVA) was used for other results. *P*<0.05 was considered statistically significant.

## RESULTS

### The platelet Aβ content increases with age

We determined the levels of the Aβ1-40 and Aβ1-42 proteins in platelets and hippocampi from two different AD mouse models at different ages to investigate the relationship between platelets and AD under natural aging conditions.

**Figure 2:**
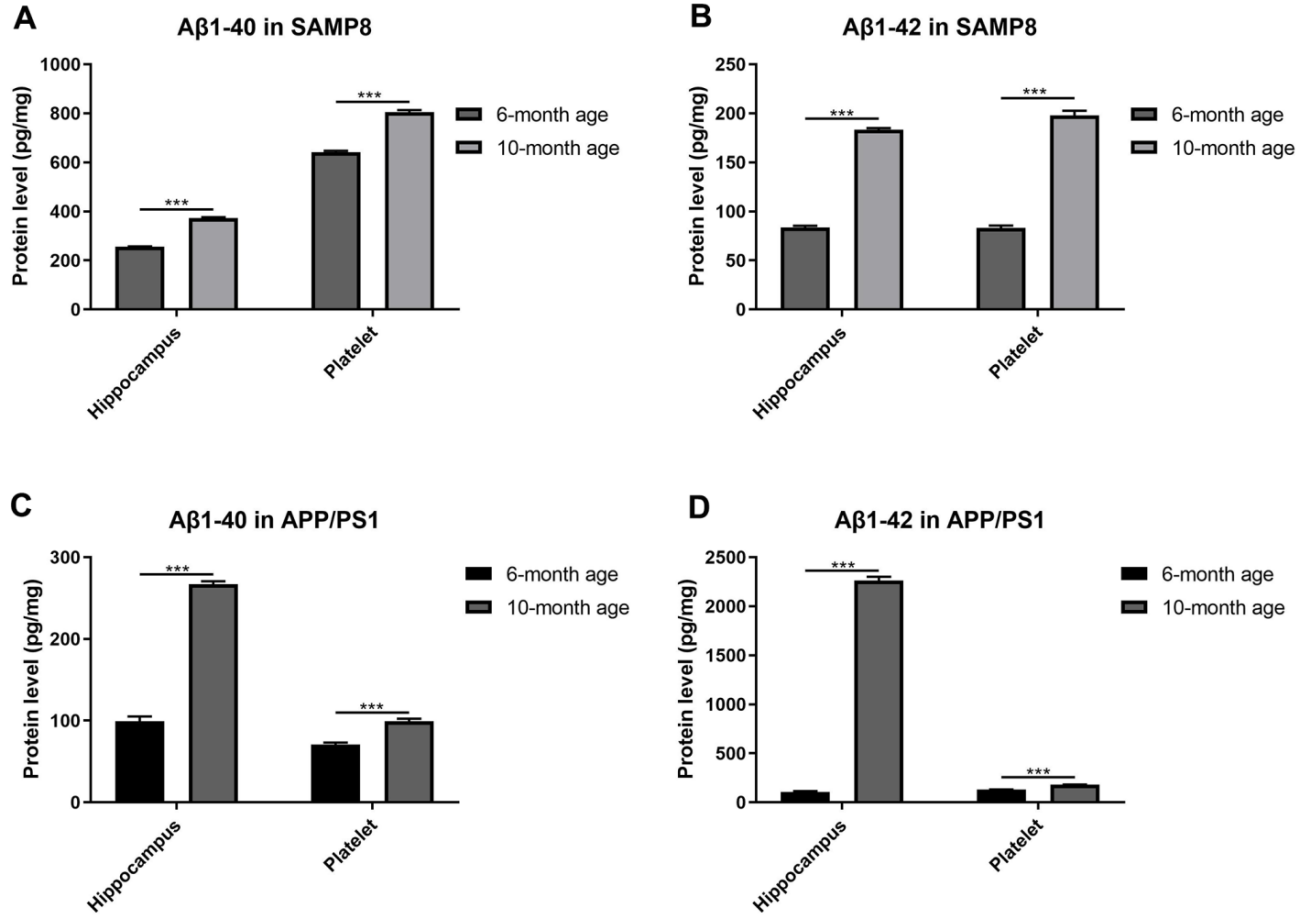
**Aβ levels in platelets and hippocampi from SAMP8 mice of different ages.** (**A**) Aβ1-40 contents in platelets and hippocampi from 6- and 10-month-old SAMP8 mice. (**B**) Aβ1-42 contents in platelets and hippocampi from 6- and 10-month-old SAMP8 mice. (**C**) Aβ1-40 contents in platelets and hippocampi from 6- and 10-month-old APP/PS1 mice. (**D**) Aβ1-42 contents in platelets and hippocampi from 6- and 10-month-old APP/PS1 mice. Each group included 5 mice, ^***^P<0.001 compared with the 6-month-old mice.

As expected, both the Aβ1-40 and Aβ1-42 contents increased in the hippocampus of SAMP8 mice (Figure 2A, 2B) and APP/PS1 mice (Figure 2C, 2D) with increasing age, suggesting that aged SAMP8 and APP/PS1 models mice shared similar pathological changes in Aβ deposition. Moreover, we observed the same trend of elevated levels of Aβ1-40 and Aβ1-42 in platelets from elderly SAMP8 and APP/PS1 mice.

### Exogenously aged APP/PS1 platelets lead to learning and memory deficits

With the aim of exploring whether platelets might be a factor contributing to AD and what type of platelets would mostly be affected, we injected platelets and plasma from APP/PS1 mice of different ages into 10-week-old C57 mice, with 4 mice in each group. IF staining was performed on brain slices from the C57 mice. As the donor mouse age increased, increased deposition of Aβ in the recipient mouse brain was observed. Additionally, the injection of platelets led to greater deposition than the plasma injection (Table 1). These results verified our conjecture about the importance of platelets in the AD process and provided an important reference for donor mouse age selection for follow-up experiments.

**Table 1:** Proportion of mice with Aβ deposition signals.

**Recipients (n=4)**	**injectant**	**Donor mouse genotype**	**Donor mouse age (months)**	**Proportion (number of mice with signals/total number of mice) (%)**
10-week old C57 mice	platelets	-/-	3	0%
	platelets	+/+	3	0%
	plasma	+/+	3	0%
	platelets	-/-	6	0%
	platelets	+/+	6	0%
	plasma	+/+	6	0%
	platelets	-/-	10	0%
	platelets	+/+	10	75%
	plasma	+/+	10	25%
	platelets	+/+	15	100%
	plasma	+/+	15	25%
	NS	-	-	0%

**Figure 3:**
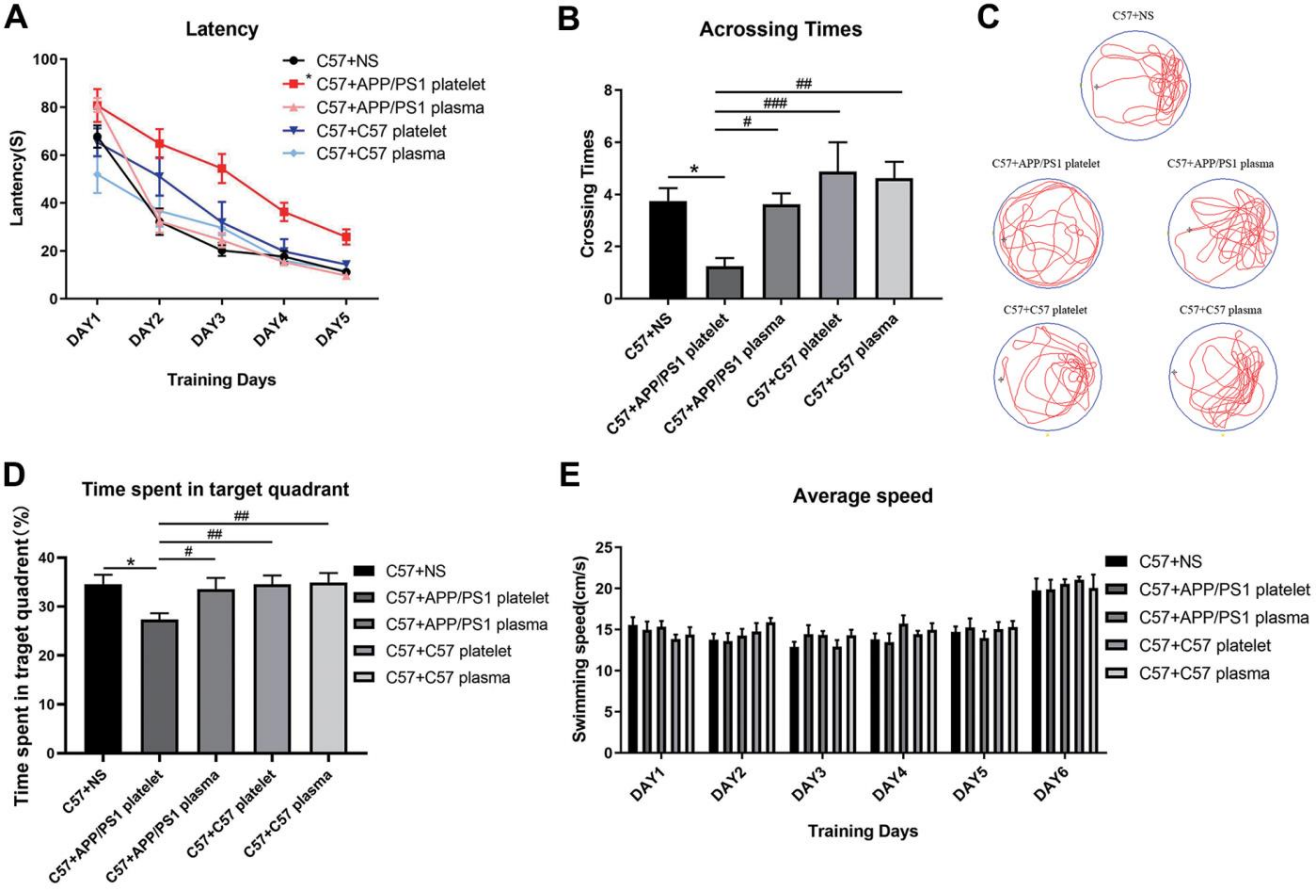
**Effects of plasma or platelets from aged mice on spatial learning and memory abilities.** (**A**) Latency (escape latency), the time the mice spent finding the underwater hidden platform on the MWM training days. (**B**) Crossing times, the number of times the mice crossed the former platform area during the probe trial on the last day. (**C**) Track trail, the track trail of the mice during the probe trial on the last day. (**D**) Time spent in the target quadrant. (**E**) Average speed, average swimming speed of the mice per day, showing that the experimental results, such as latency, are not related to the swimming speed of the mice. Each group contained 8 mice. ^*^P<0.05 compared with C57+NS; ^#^*P*<0.05, ^##^*P*<0.01, and ^###^*P*<0.001, compared with C57+APP/PS1 platelets.

Subsequently, we chose 15-month-old APP/PS1 mice as donors to provide platelets and plasma, 15-month-old C57 mice as negative control donors, and saline as a blank control. We performed the MWM test to evaluate learning and memory abilities. The group injected with platelets from APP/PS1 mice required an increased latency to find the hidden platform compared with the saline-treated mouse group, while the other 3 groups did not show a significant reduction in latency time (Figure 3A). Similarly, the number of crossings over the platform site was significantly reduced in the group injected with platelets from APP/PS1 mice compared with the other three groups of mice (Figure 3B). Furthermore, a marked difference in the track map of the probe test was observed among the treated groups (Figure 3C). Additionally, a significant decrease in the total time spent in the target quadrant was observed in the APP/PS1 platelet-treated mouse group compared with the other groups (Figure 3D). The average daily speed of each group was not significantly different, which supports the conclusions described above (Figure 3E).

### Exogenously aged APP/PS1 platelets increase the level of the AD marker protein

We performed IF staining on brain slices to verify whether injected exogenous platelets affect the deposition of Aβ in the brains of recipient mice. The injection of platelets from aged APP/PS1 mice through the tail vein led to Aβ aggregation and microglial activation in the hippocampus. However, plasma from aged APP/PS1 mice and platelets and plasma from aged C57 mice did not cause Aβ aggregation (Figure 4A). The measurements of the fluorescence intensity produced similar results (Figure 4B, 4C).

**Figure 4:**
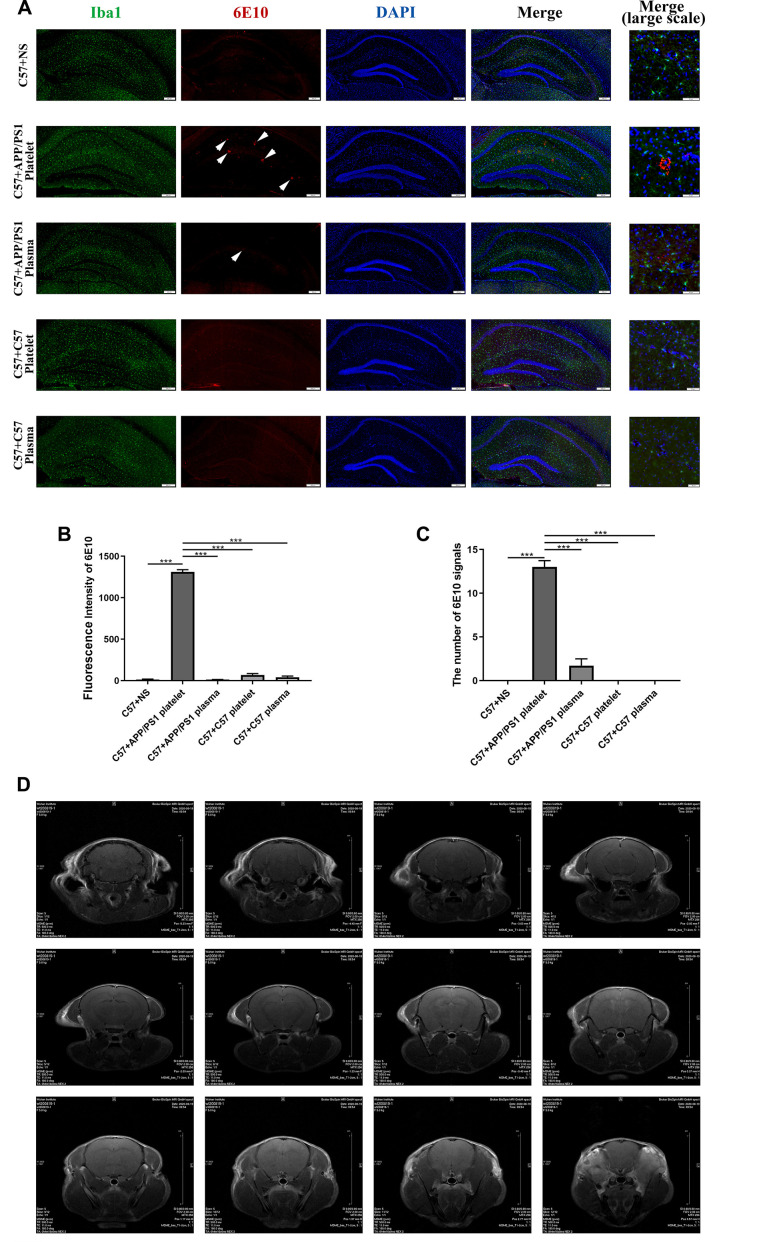
**Effects of tail vein injection of plasma or platelets on AD related indexes of blood and brain tissue in mice.** (**A**) IF staining of brain slices with anti-Iba1 (green) and anti-6E10 (red) antibodies, and the nucleus was stained with DAPI (blue). The arrow indicates the expression of 6E10, which labels the Aβ protein. (**B**) Fluorescence intensity of 6E10. (**C**) The number of 6E10 signals. Each group contained 8 mice, and 6 similar coronal sections were selected from each brain for IF staining. Scale bars: high-magnification images = 50 μm, other images = 200 μm. ^***^P<0.001 compared with C57+APP/PS1 platelets. (**D**) Brain image of a C57 mouse by MRI.

**Figure 4:**
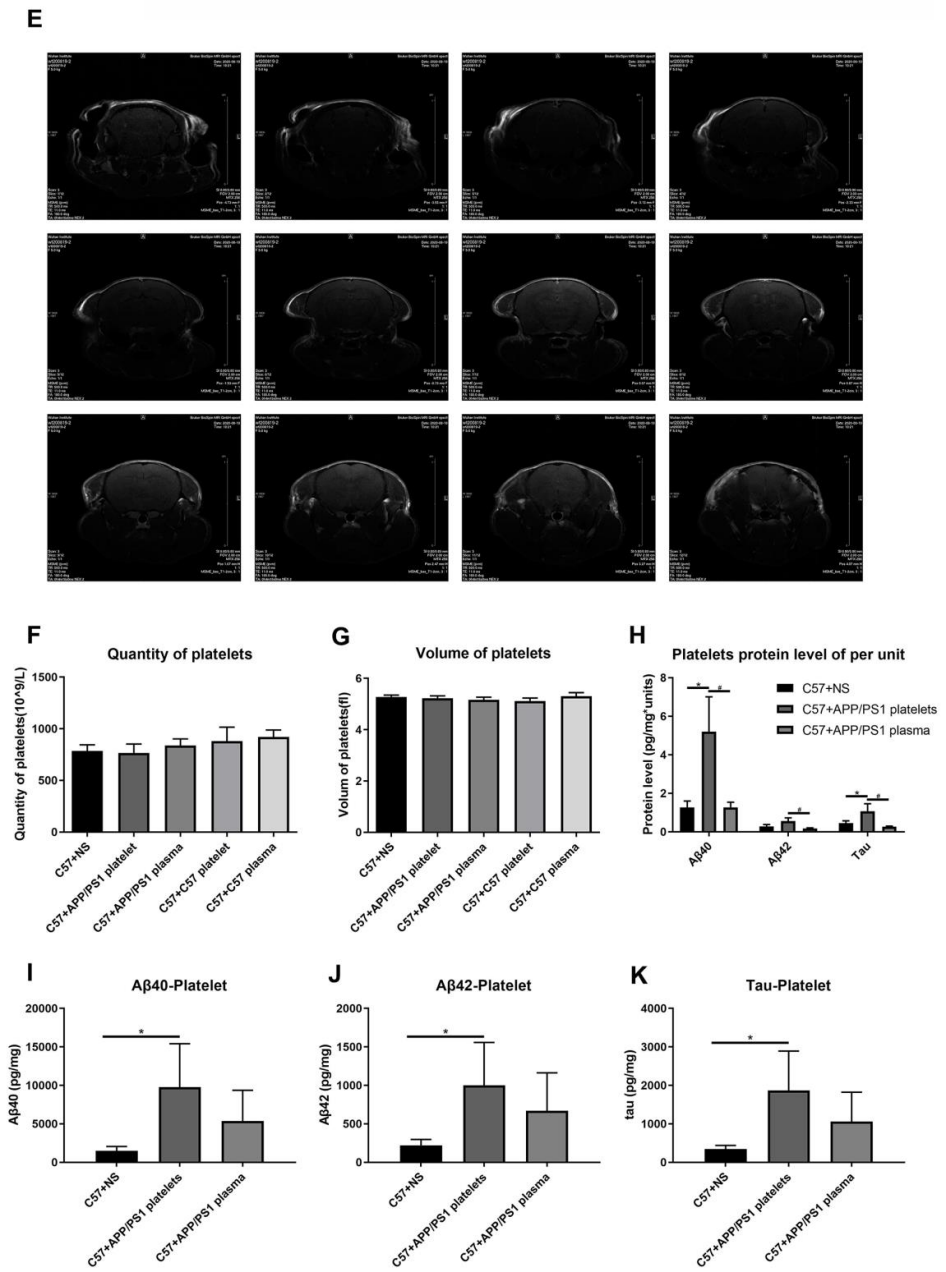
**Effects of tail vein injection of plasma or platelets on AD related indexes of blood and brain tissue in mice.** (**E**) Brain image of a C57+APP/PS1 platelet mouse by MRI. (**F**) Quantity of platelets. (**G**) Volume of platelets. Each group included 8 mice. No significant differences were observed between groups. (**H**) Aβ1-40, Aβ1-42 and tau protein levels per unit of platelets. (**I**) Aβ1-40 levels in platelets. (**J**) Aβ1-42 levels in platelets. (**K**) Tau levels in platelets. Each group included 8 mice. ^*^P<0.05 compared with C57+NS and ^#^*P*<0.05 compared with C57+APP/PS1 platelets.

We also conducted magnetic resonance imaging (MRI) experiments to determine damage to the BBB by detecting contrast agents in the mouse brain. However, this experiment did not reveal any obvious pathological damage (Figure 4D, 4E).

A routine blood examination of the platelets indicated no significant differences between each treatment group, and the intervention of injecting platelets or plasma through the tail vein did not affect the normal physiological conditions of peripheral blood in the mice (Figure 4F, 4G). The ELISAs of levels of the Aβ1-40, Aβ1-42 and tau proteins in platelets indicated that the injection of the exogenous blood components from aged APP/PS1 mice increased the levels of the Aβ and tau proteins, and the platelet injection increase levels of the Aβ and tau proteins to a greater extent than the plasma injection (Figure 4I–4J). Similarly, the protein level in platelets per unit of platelets exhibited a similar trend (Figure 4H).

### Aspirin rescues memory deficits and decreases the expression of AD marker proteins in SAMP8 mice

Based on the results we obtained, we wished to further assess whether platelet activity plays an important role in the AD process. We chose SAMP8 mice to simulate the situation of natural aging, and SAMR1 mice as the normal control to explore the protective effects of ASA on inhibiting platelet activity during the aging process.

**Figure 5:**
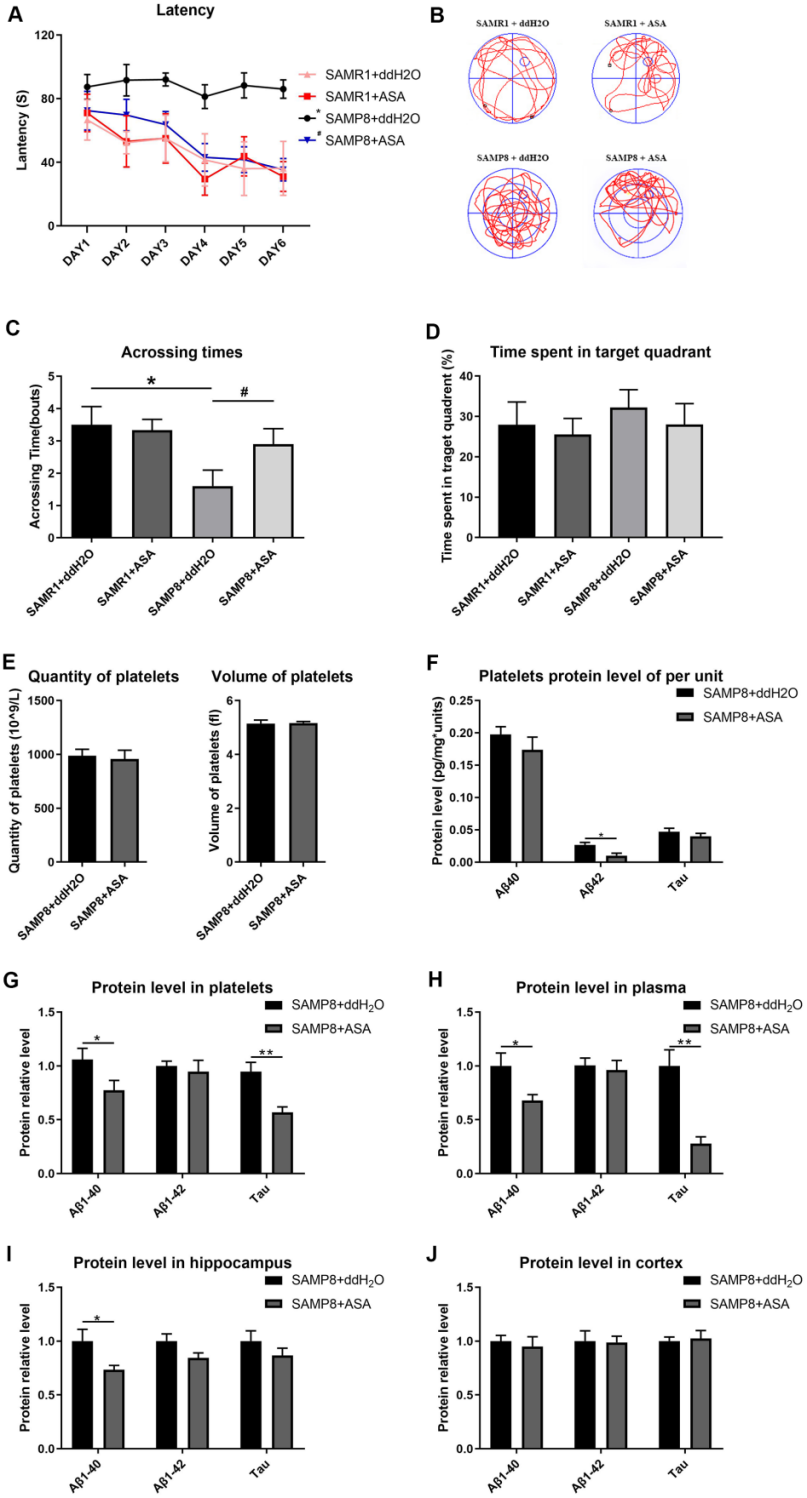
**Effects of intragastric administration of ASA on learning and memory ability and AD related indexes of blood and brain tissue in mice.** (**A**) Latency (escape latency), the time mice spent finding the underwater hidden platform in the MWM on training days. (**B**) Track trail, mice track trail during the probe trial on the last day. (**C**) Crossing times, the number of times the mice crossed the former platform area during the probe trial on the last day. (**D**) Time spent in the target quadrant. n=6 in the SAMR1+ddH_2_O and SAMR1+ASA groups; n=10 in the SAMP8+ddH_2_O and SAMR1+ASA groups. ^*^P<0.05 compared with the SAMR1+ddH_2_O group and ^#^*P*<0.05 compared with the SAMR1+ASA group. (**E**) The quantity and volume of platelets. (**F**) Aβ1-40, Aβ1-42 and tau protein levels per unit of platelets. (**G**) Aβ1-40, Aβ1-42 and tau protein levels in platelets. (**H**) Aβ1-40, Aβ1-42 and tau protein levels in plasma. (**I**) Aβ1-40, Aβ1-42 and tau protein levels in the hippocampus. (**J**) Aβ1-40, Aβ1-42 and tau protein levels in the cortex. ^*^P<0.05 and ^**^P<0.01 compared with the SAMP8+ddH_2_O group.

The results of the MWM test indicated that SAMP8 mice treated with ASA exhibited a decreased latency to find the hidden platform compared with the untreated SAMP8 mice (main effect *P*<0.001). In the two SAMR1 groups, treatment with ASA did not exert any effect compared to the normal group (Figure 5A). Similarly, the number of crossings over the platform site was significantly increased in the SAMP8 mice treated with ASA, and was approximately the same as the SAMR1 groups (Figure 5C). Furthermore, a significant difference in the track map of the probe test was observed among the treated groups (Figure 5B). The time spent in the target quadrant by each groups of mice also exhibited the same tendency (Figure 5D).

A routine blood examination of the platelets indicated that no statistically significant differences between each treatment group, indicating that ASA gavage treatment did not affect the normal physiological condition of the mice (Figure 5E). Thus, ASA reduced platelet Aβ secretion, as a tendency toward reduced levels of the Aβ and tau proteins was observed in plasma, platelets and the hippocampus (Figure 5F–5J).

### Exogenously aged APP/PS1 platelets change the biological characteristics of the *in vitro* BBB model

**Figure 6:**
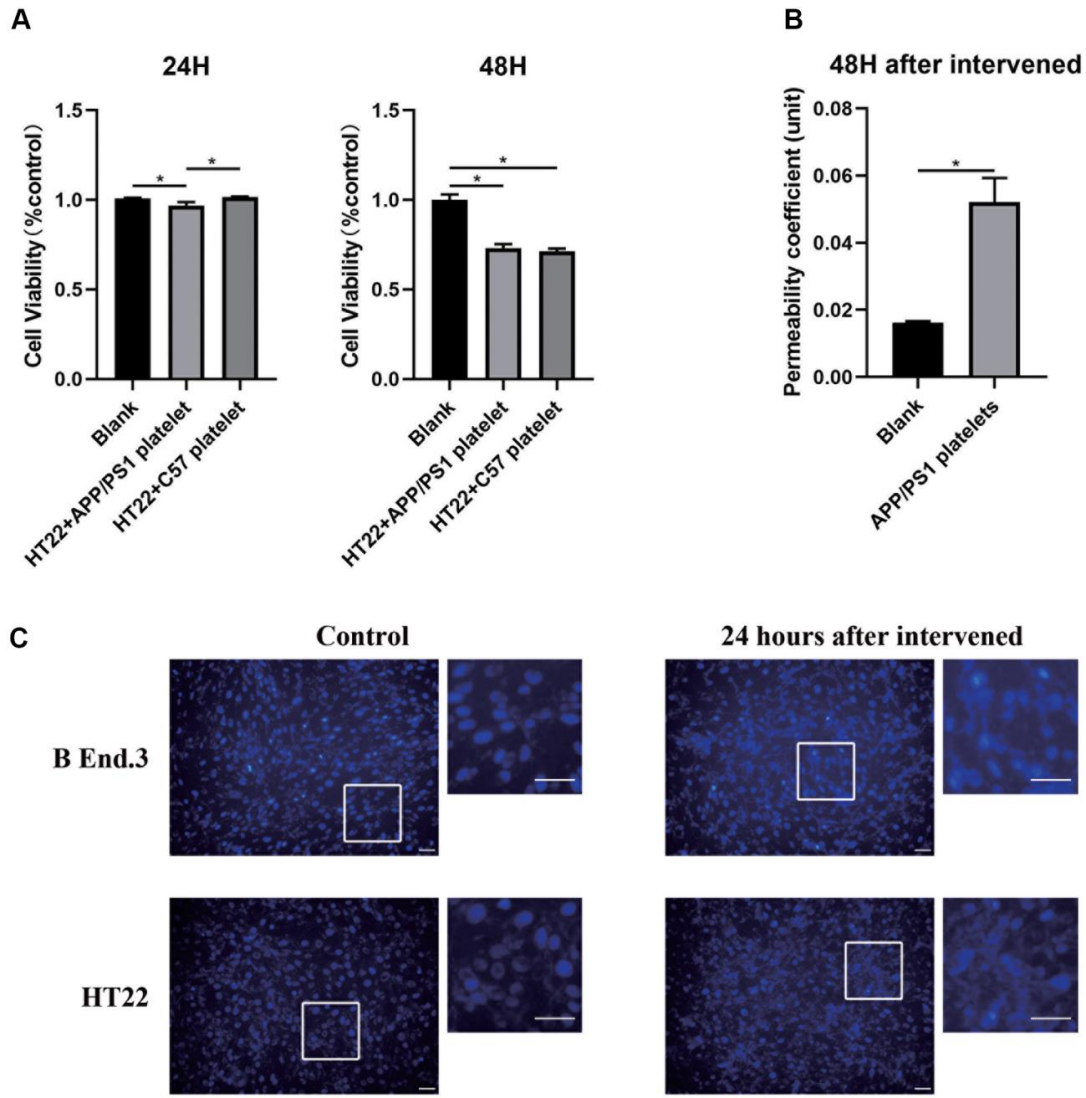
**Alteration of the *in vitro* BBB model after the platelet intervention.** (**A**) Cell viability analyzed using the CCK-8 assay. (**B**) Permeability of the *in vitro* BBB model. (**C**) Apoptosis assay with Hochest-33342 staining. Scale bar=100 μm.

We designed an *in vitro* model of the BBB by referring to previous studies to explore the cellular mechanisms by which platelets may affect the BBB. We first evaluated the cell apoptosis level after treatment with platelets from aged APP/PS1 mice using the CCK-8 assay and Hoechst-33342 staining. The results illustrated different degrees of apoptosis, the occurrence of nucleation, and a decrease in the cellular activity of both in b End.3 and HT22 cells (Figure 6A, 6C). We measured the permeability of the BBB in an *in vitro* model before and after treatment with platelets, and found that the permeability increased after the intervention (Figure 6B). Therefore, the platelets damaged the biological characteristics of the *in vitro* BBB model.

## DISCUSSION

Researchers been increasingly interested in Aβ derived from peripheral blood and its effects on the central nervous system. The results of our previous study also suggested that platelets may provide a new insight into the pathogenesis of AD [33–35]. Nakamura et al. reported that peripheral plasma contains biomarkers that predict the levels of Aβ in the brain and provided a method for the early diagnosis of AD [36]. According to a study by Scudellari, a transfusion of blood or plasma from young individuals into older patients effectively alleviates AD symptoms [37]. In addition, Aβ deposition in the brain may potentially correlate to the level in the peripheral circulatory system [14]. Based on these findings, platelet-derived Aβ might affect the central nervous system. In our experiments, we found that platelets may accelerate the AD process by disrupting the permeability of the BBB and accelerating the accumulation of Aβ. In addition, ASA appears to exert certain protective effects on the process of AD.

Platelet-derived Aβ must cross the BBB before forming plaques and deposits in the brain. Bu et al. suggested that blood-derived Aβ may enter the brain through the BBB, and then cause neuroinflammation, degeneration and hippocampal CA1 long-term potentiation in the brain [38]. APP/PS1 mice are double-transgenic mice that express mutant human presenilin and human murine amyloid proprotein fusion proteins, and are often used to explore the role of APP in AD. In our study, we illustrated that platelets from aged APP/PS1 mice injected through the tail vein led to learning and memory deficits. Compared with the two groups with the same donors, the platelet-injected group exhibited a more pronounced decrease in learning and memory and increased Aβ deposition than the mice injected with plasma. Consequently, Aβ produced by platelets from aged APP/PS1 mice entered the brain through the BBB and then was deposited in the hippocampus to potentially participate in the course of AD. The experiments reported by Bu emphasized changes in neuroinflammatory factors in the brain [38], while our experiments focused on behavioral research, reflecting alterations in cognitive and learning abilities in experimental mice administered platelets. Our results further validate the speculation that platelets are important in the AD process.

Moreover, Festoff et al. identified endothelial cell damage in the BBB as a potentially important factor in the promotion of AD [39]. Nation et al. found higher BBB permeability in patients with AD than in people without AD, and thus peripheral blood components are more likely to affect the stable environment of the brain and cause inflammation and neurotoxicity [40]. Therefore, we designed an *in vitro* model of the BBB by referring to a previous study to verify how the Aβ secreted by platelets affects intracranial nerve cells across the BBB [41]. Platelets from aged APP/PS1 mice reduced the viability of HT22 neurons, while increasing the number and degree of apoptotic b End.3 epithelial cells and HT22 neurons. The permeability of the BBB in the model also increased due to epithelial cell damage. However, the MRI results did not confirm this change in permeability. This result may be because the contrast agent did not act for a sufficient time or the degree of damage was not sufficiently severe to be detected. Thus, further verification and analysis in follow-up experiments are required. From these results, we speculate that vascular endothelial cell injury and abnormal BBB function might be factors that lead to the entry of peripheral Aβ into the brain.

Here, we explored whether we could intervene in the process of Aβ entry into the brain that causes cognitive impairment. Aβ is released into plasma when platelets aggregate; conversely, the inhibition of platelet aggregation reduces Aβ release [42]. ASA, an inhibitor of platelet aggregation was used as a platelet activity inhibitor to evaluate the effects of platelets on the AD process in many studies. Its effect has always been controversial, some researchers believed that there was no clinical evidence that aspirin was effective in reducing risk of dementia, MCI, or cognitive decline [43]. But some researchers have confirmed through cell-based and animal experiments that inflammation is a notable factor contributing to AD pathogenesis. The anti-inflammatory effects of ASA might have affect in AD progression, because ASA prevents and improves mitochondrial dysfunction in cells and might be a potential research object for AD treatment [44–46]. We observed the behavioral effects of ASA on AD animal models to further verify whether ASA improved the cognitive function and learning ability of elderly SAMP8 mice. These SAM mice were selectively inbred and presented signs of senility under normal physiological condition, providing a more suitable model for exploring aging related diseases than the APP/PS1 mice mentioned above [28, 29]. In our experiment, ASA exerted a certain effect on the cognitive function of SAMP8 mice in the MWM exploration experiment, with results that were similar to the SAMR1 normal control mice. Subsequently, we examined the changes in the levels of the Aβ1-40, Aβ1-42 and tau proteins in the platelets, plasma, the hippocampus and cortex of SAMP8 mice using ELISAs. As expected, ASA reduced the concentrations of Aβ1-40, Aβ1-42, and tau in both platelets and plasma. More importantly, ASA significantly reduced the Aβ1-40 protein content in the hippocampi of mice, which was accompanied by a tendency to reduce the deposition of Aβ1-42 and tau proteins. Changes in the levels of these proteins in the brain correlated with the changes in peripheral platelets and plasma, consistent with improvements in the cognitive functions of the mice. Our results indicated that ASA exerted a positive protective effect on the ageing process of SAMP8 mice, such as decelerating learning and memory dysfunction and reducing the secretion of AD marker proteins. Furthermore, a recent study by Sepulveda et al. revealed that Aβ is regulated by the cAMP/PKA pathway in human platelets, and an increase in cAMP hinders the processing and secretion of Aβ [47]. ASA increases the cAMP level in rat platelets after stimulation with ADP [48]. These results might explain the mechanism by which ASA reduces the concentrations of Aβ1-40 and Aβ1-42 in platelets.

## CONCLUSIONS

Based on our studies, allogeneic platelets from AD model mice increased the deposition of Aβ in the recipient’s brain, induced learning and memory deficits in normal young mice, and to a certain extent, damaged the BBB of mice and increased its permeability, revealing that platelets might be a culprit that leads to AD. However, these processes were reversed by the ASA treatment, indicating that ASA deserves further investigation in the treatment of AD.

### Ethical approval and consent to participate

All animal experiments were authorized by the Animal Ethics Committee of Sun Yat-sen University (SYSU-IACUC-2019-B990).

### Availability of data and materials

All raw data used and/or analyzed during the current study are available from the corresponding author upon reasonable request.
